# Design and implementation of an end-to-end AI-driven colonoscopy recall workflow at scale

**DOI:** 10.1093/jamiaopen/ooag070

**Published:** 2026-05-06

**Authors:** Aman Mohapatra, Rachel Porth, Si Wong, Heather Hardway, Gail Piatkowski, John Shang, Maelys J Amat, Sarah Flier, Adam Salsman, Ted Fitzgerald, Ayad Shammout, David Rubins, Amy Miller, Venkat Jegadeesan, Arvind Ravi, Joseph D Feuerstein

**Affiliations:** Department of Medicine, Beth Israel Deaconess Medical Center, Harvard Medical School, Boston, MA, 02215, United States; Department of Medicine, Beth Israel Deaconess Medical Center, Harvard Medical School, Boston, MA, 02215, United States; Information Systems, Beth Israel Lahey Health, Boston, MA, 02215, United States; Halo Solutions, LLC, Boston, MA, 02215, United States; Information Systems, Beth Israel Lahey Health, Boston, MA, 02215, United States; Information Systems, Beth Israel Lahey Health, Boston, MA, 02215, United States; Department of Medicine, Beth Israel Deaconess Medical Center, Harvard Medical School, Boston, MA, 02215, United States; Department of Medicine, Beth Israel Deaconess Medical Center, Harvard Medical School, Boston, MA, 02215, United States; Information Systems, Beth Israel Lahey Health, Boston, MA, 02215, United States; Information Systems, Beth Israel Lahey Health, Boston, MA, 02215, United States; Information Systems, Beth Israel Lahey Health, Boston, MA, 02215, United States; Department of Medicine, Beth Israel Deaconess Medical Center, Harvard Medical School, Boston, MA, 02215, United States; Information Systems, Beth Israel Lahey Health, Boston, MA, 02215, United States; Department of Medicine, Beth Israel Deaconess Medical Center, Harvard Medical School, Boston, MA, 02215, United States; Information Systems, Beth Israel Lahey Health, Boston, MA, 02215, United States; Information Systems, Beth Israel Lahey Health, Boston, MA, 02215, United States; Halo Solutions, LLC, Boston, MA, 02215, United States; Department of Medicine, Beth Israel Deaconess Medical Center, Harvard Medical School, Boston, MA, 02215, United States

**Keywords:** artificial intelligence, large language models, electronic health records, natural language processing, colorectal cancer screening, data migration

## Abstract

**Objectives:**

To develop a large-language-model (LLM)–centric workflow flow extraction and migration of clinician-documented colonoscopy recall recommendations from unstructured reports and letters during an enterprise-wide electronic health record (EHR) transition.

**Materials and Methods:**

A multi-stage workflow [Optical Character Recognition (OCR) -> LLM -> structured fields] was built around a central GPT-4 Turbo inference step following prompt optimization. Validation was performed on a held-out set (*N* = 326 notes) using 2-clinician consensus and then benchmarked against traditional rule-based natural-language-processing (NLP) (spaCy v3). Layered quality control—manual review, field validation, and anomaly detection—was used to assess workflow results prior to upload (*N* = 118 181 total patients).

**Results:**

Prompt optimization enabled GPT-4 Turbo to achieve perfect concordance with clinician review in a small test set (macro-F1 = 1.0; *N* = 100 patients). Expanded validation on a held-out set demonstrated improved F1 (0.89; CI = [0.65, 0.92], *N* = 326) relative to a traditional rule-based NLP approach (F1 = 0.78; CI = [0.58, 0.82]). The system processed 118 181 records in ≈9 hours (≈2 s/record) at a direct implementation cost of ∼$12 000.

**Discussion:**

An LLM-driven workflow safely migrated preventive-care data at population scale, with potential accuracy improvements over traditional rule-based NLP approaches and substantial reductions in time and cost relative to manual review.

**Conclusion:**

LLMs can play a valuable role in high-quality structuring of clinical data, preserving longitudinal care continuity during EHR modernization.

## Background and significance

Healthcare digitization has shifted towards structured data capture, enabling precise retrieval, automation, and analytics.^1^ Electronic Health Records (EHRs) increasingly rely on structured fields to support quality measures, guideline adherence, and clinical decision support tools.^2^ This shift has enabled measurable improvements in data standardization and care coordination but has also highlighted limitations in legacy documentation systems during large system data transitions.^3^

More specifically, existing free-text clinical data may not be retrospectively structured at scale, leaving critical information inaccessible. Manual abstraction to restore such information is often impractical; chart-review studies estimate up to 30 minutes per record.^4^ Transitions between EHR systems are recognized patient-safety vulnerabilities because structured “health-maintenance” fields such as colonoscopy recall intervals—the recommended time frame in which a patient should return for their next colonoscopy—rarely migrate intact, leading institutions to default to generic intervals rather than individualized recommendations. This creates a transitional period of clinical risk: clinicians assume structured fields are accurate, but historical data may be misrepresented. Prior to our institution’s EHR transition, many patients would have received default recall values rather than previously documented patient-specific recommendations.^3^ When structured health maintenance fields are relied upon to track surveillance intervals, errors in data migration can result in incorrect surveillance.

Prior research has shown that colonoscopy and pathology narratives contain enough structured cues to automatically determine appropriate surveillance intervals. For example, one study developed a rule-based natural-language-processing (NLP) and decision-support system that generated guideline-concordant recall intervals, while another study extended this approach using a hybrid NLP/statistical model that achieved approximately 92% accuracy compared to manual gastroenterologist review.^5^^,^^6^ These studies established the feasibility of automated interval extraction but depended on highly templated reports and consistent documentation structures, limiting scalability across heterogeneous real-world data.

**Figure 1.**
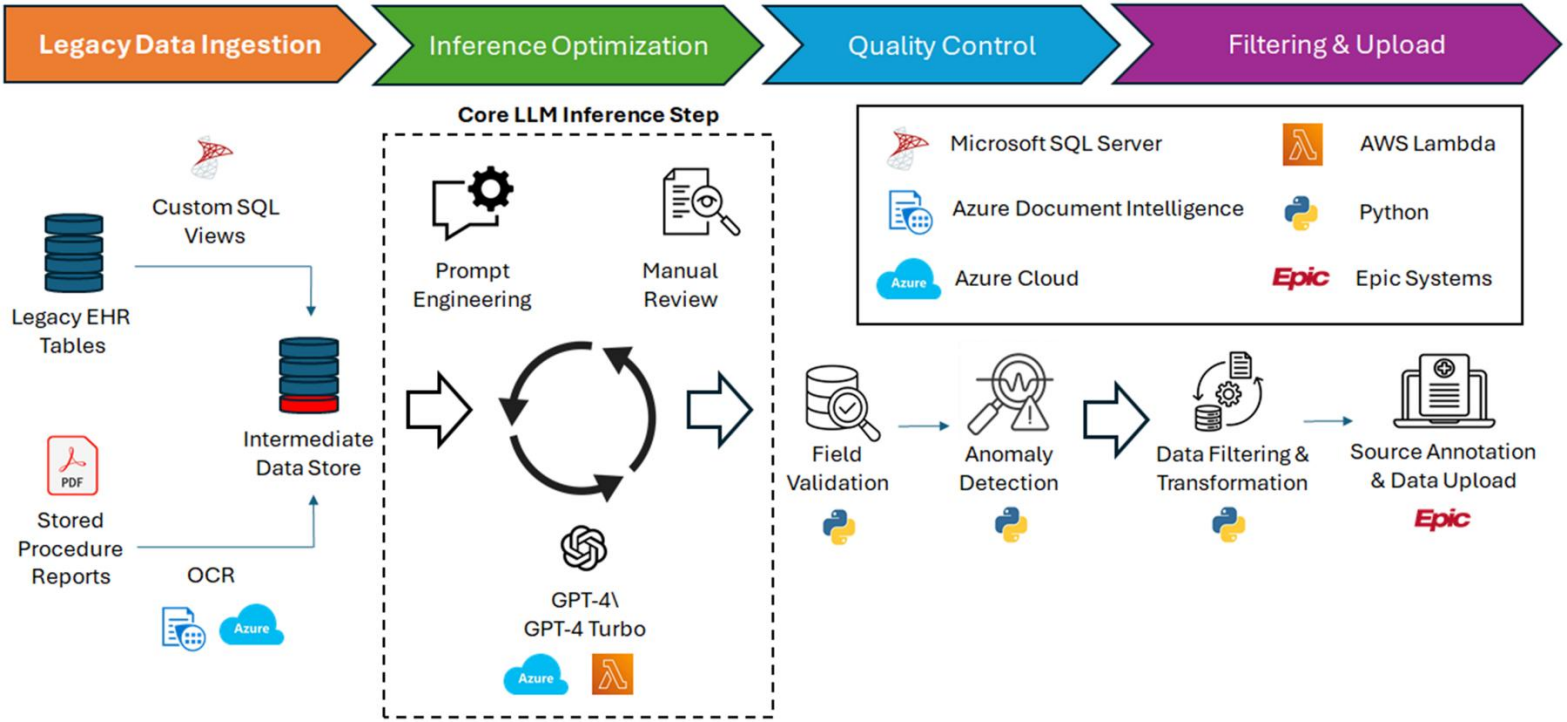
Overview of end-to-end LLM-driven colonoscopy recall pipeline. The recall pipeline development process involved 4 stages, namely (1) legacy data ingestion, in which primary legacy EHR tables were extracted via custom SQL views and merged with stored procedure reports which were separately processed via an OCR pipeline involving Azure Document Intelligence; (2) inference optimization, which is the traditionally emphasized core LLM inference step involving iterations of prompt design, inference, and manual review; (3) quality control, using a combination of field validation and anomaly detection to identify and process inference results at scale; and (4) filtering and upload, in which inference results were transformed into an upload ready format with the addition of a comment citing the source clinical document (ie, provenance annotation) to aid physician review. Abbreviations: AWS, Amazon web services; EHR, electronic health record; OCR, optical character recognition; SQL, structured query language . Created by authors using Microsoft Powerpoint with icons sourced from royalty-free image repositories (eg, Google Images/Flaticon). No copyrighted content was used.

To accurately facilitate data migration during our institution’s EHR transition, we developed an end-to-end LLM-centric system to extract, validate, and populate colonoscopy recall fields using historical procedural documentation. Our pipeline identified clinically appropriate recall intervals from unstructured reports (with improved performance over traditional rule-based NLP), flagged errors and edge cases, and accurately transferred colonoscopy recall data to the new EHR (Figure 1). In doing so, the system preserved clinician intent by migrating documented recommendations rather than inferring guideline-based intervals—emphasizing information capture and migration, not decision support.

## Objective

The objective of this real-world quality improvement study was to design, implement, and evaluate a large-language-model (LLM)–based informatics pipeline that accurately extracts and migrates clinician-documented colonoscopy recall recommendations from unstructured legacy data during an enterprise-wide EHR transition. Specifically, the study aimed to assess whether GPT-4 Turbo could accurately infer recall intervals from historical endoscopy reports and patient letters, benchmarking its performance against a traditional rule-based NLP baseline, and populate structured health maintenance fields in the new EHR with minimal clinician oversight. In addition to evaluating model accuracy against clinician annotation, the study assessed the performance of multilayered quality-control strategies—including field validation, anomaly detection, and automated provenance tagging—to ensure fidelity and reliability at population scale. A secondary objective was to perform an exploratory modeling analysis of the potential operational, clinical, and economic implications of replacing default recall intervals with patient-specific recommendations during EHR modernization.

## Methods

### Project initiation and approvals

Key stakeholders—including leadership from hospital administration, information systems, gastroenterology, and quality and safety teams—collaboratively designed an optimal AI-driven recall workflow to transition from our legacy EHR (webOMR) to Epic aligning with institutional quality-improvement governance and IRB exemption procedures.

### Preprocessing of legacy data

Custom SQL queries generated views from our legacy enterprise data warehouse consisting of 118 181 patients for patient demographics, endoscopy scheduling, and clinical notes (2014-2024). Historical documents included both patient letters, which summarized procedure and pathology findings, and scanned procedure reports, which required Optical Character Recognition (OCR) preprocessing. OCR was performed using Azure Document Intelligence (Layout Model) to convert PDF reports into structured text prior to inference.

When both document types were available for a patient, the letter was prioritized because it typically contained the finalized recall interval incorporating pathology results.

### Optimization of LLM-based recall inference

We used AWS lambda to test prompts for extracting recall intervals from free text inputs using GPT-4 (gpt-4-0314) and GPT-4 Turbo (gpt-4-1106-preview). When available, recall from patient letters was prioritized as these typically integrate both endoscopy and pathology findings. An initial test set of 100 patients was used for iterative prompt optimization, followed by a set of *N* = 20 challenging edge cases. F1 scores were calculated against manual clinician review.

### Prompt design and output formation

The LLM prompt was optimized iteratively to ensure consistent field extraction across varying note structures. To standardize outputs for downstream validation, each inference was requested in JSON format:

{"recall_interval_value": X, "unit": "years|months," "source": "letter|report"}.

This schema simplified quality control by allowing automated filtering for invalid, null, or implausible results. F1 scores were calculated against manual clinician review, with precision, recall, and 95% CIs reported.

### Gold standard and validation

Two board-certified clinicians independently annotated a held-out validation set of 326 patient notes to establish a gold standard for recall intervals (sampled uniformly across our study interval but filtered to exclude patients used for prior prompt optimization). Discrepancies were adjudicated by consensus. Model outputs were compared to this clinician reference using precision, recall, and F1-score metrics with 95% CIs. Inter-rater agreement between reviewers was calculated using Cohen’s *κ* statistic. Invalid or null values identified during validation were flagged for prompt revision before scaling the full dataset.

### Baseline NLP comparison

To benchmark performance, we implemented a rule-based NLP baseline using spaCy v3 Named-Entity Recognition to identify interval expressions (eg, “in 3 years,” “repeat at age 50”) within a comparison cohort (*N* = 21 172). Extraction outputs were stratified by agreement of between the 2 models and by clinical severity of deviation: minor (≤6 months), moderate (>6 months to ≤2 years), and major (>2 years). Outputs were evaluated within each stratum to adjudicate the accuracy of each model’s inference, enabling comparison of model performance across increasing categories of downstream clinical risk.

To characterize the nature of clinically meaningful discrepancies relevant to deployment, spaCy v3 errors within clinically significant strata (moderate and major deviation) were further manually reviewed and categorized into 3 primary failure modes: collapse of interval ranges or conditional surveillance recommendations, failure to compute age-anchored or date-dependent recall timing, and context confusion in which non-recall timing was extracted. Failure mode frequencies were estimated using stratified bin-weighted sampling, calculated as the proportion of each failure mode within a given stratum multiplied by the prevalence of that stratum in the full inference cohort and summed across strata.

### Quality control and anomaly detection

We employed 3 complementary approaches for quality control:

1. **Manual review**: For initial prompt engineering and small-scale assessment (*N* ≤ 100), we assessed discordance from clinician judgment.
2. **Field validation**: For intermediate and large-scale data, we summarized unique outputs for each component and assessed for invalid results and outliers (eg, “PARSE ERROR,” null, or out-of-range intervals).
3. **Anomaly detection**: For large-scale review, we derived additional features from primary inputs/outputs (eg, “age at last colonoscopy,” “age at recall”) and assessed to flag clinically implausible values (<6 months or >10 years) or patients beyond surveillance age.

Together, these layers allowed scalable validation when manual review was no longer feasible, uncovering rare but correctable inference errors occurring approximately once per 1000–10 000 cases.

### Postprocessing and data upload

Based on quality control assessments, we performed final filtering using custom Python scripts. For transparency, we included an automatically generated comment indicating the source and date of recommendations. The recall list was saved in CSV format for bulk import (Chronicles Import Utilities).

Validated outputs were formatted for Epic’s health maintenance module, including a provenance note (“AI-extracted recall from report on MM/DD/YYYY—please confirm”) to facilitate clinician verification.

### Workflow automation

The pipeline was established as a batch process run in multiple phases: (1) first for testing and development, (2) population scale deployment 2 months prior to go-live, and (3) a final pre–go-live run to capture interim colonoscopy results.

## Results

### Inference optimization and model selection

As a first step in optimizing our core LLM-based inference step, we began by experimenting with simple prompts to extract structured recall data from procedure reports and patient letters. Because recalls are sometimes specified as ranges, we attempted to extract data as a lower and upper bound (rather than a single value), and with fields for units (eg, months, years) to accommodate short recall periods. These and future adjustments to the prompt text (eg, note title parsing, etc.) are shown in Figure S1.

**Figure 2.**
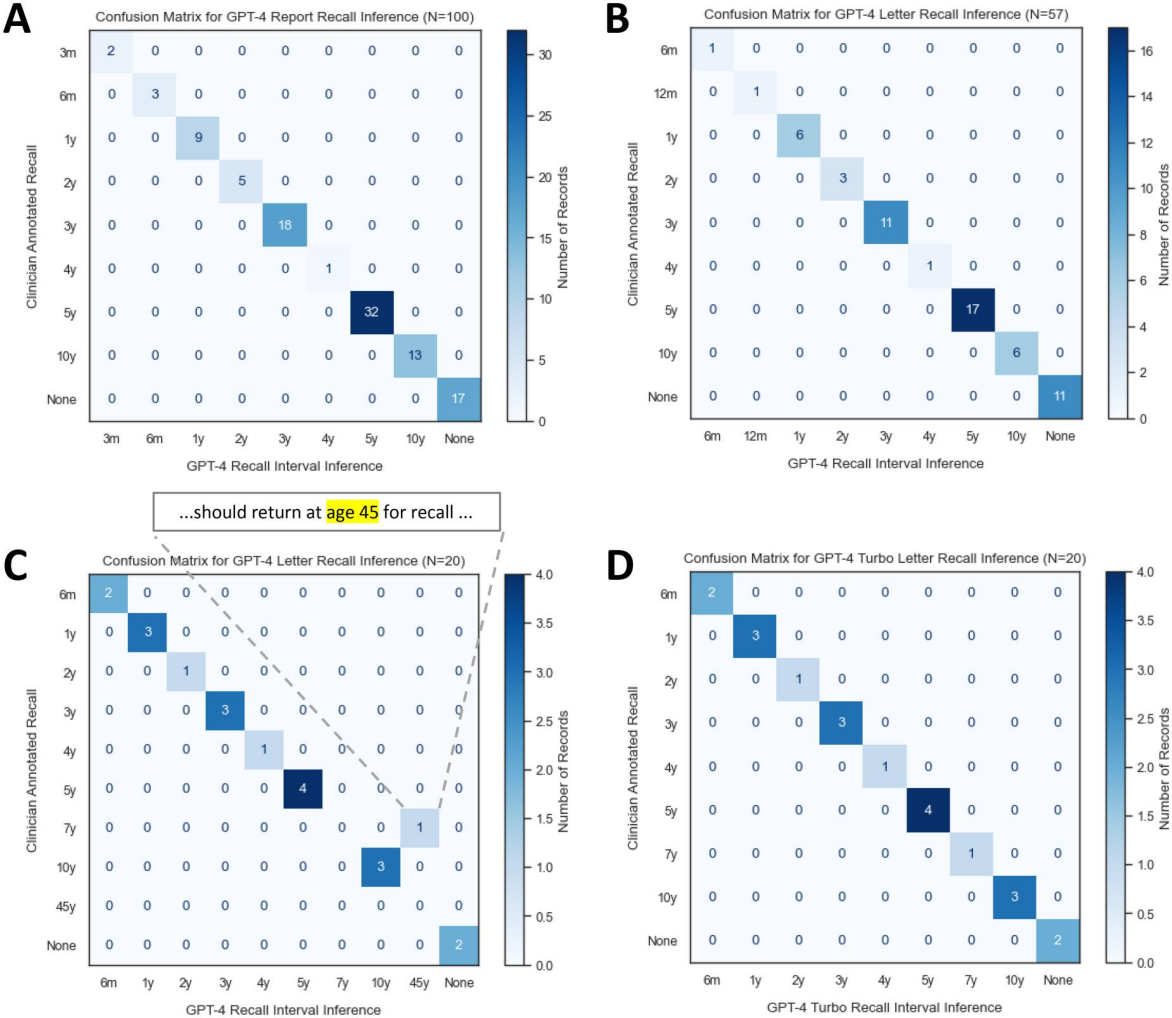
(A-D) Confusion matrices for LLM GPT-4/GPT-4 Turbo inference performance compared to clinician review. Following optimization from smaller scale testing, results from formal assessment of performance of procedure reports (A) and patient letters (B) for *N* = 100 patients showed perfect concordance with manual clinician assessment (Macro F1 = 1.0). (C) Testing on a collection of 20 edge cases with dual colonoscopy and EGD recall demonstrated a failure mode of GPT-4 in which age at recall (45 years old) was misinterpreted as recall interval (45 years). (D) Further prompt refinement and switching from GPT-4 to GPT-4 Turbo was able to rescue this failure while maintaining high performance on previously annotated data (confirming lack of performance regression). Original figure created by authors using data from hospital database (refer to methods section). Visualization designed using Adobe Illustrator.

To formally assess the performance of GPT-4 using our updated prompt, we performed inference on procedure reports and patient letters from an unseen sample of 100 patients. The model showed excellent performance on this initial assessment, with perfect concordance relative to manual clinician annotations (Macro F1 = 1.0; 95% CI [0.78-1.0]; Figure 2A and B).

To better explore performance on potential edge cases, we performed subsequent testing on a more challenging set of 20 cases from bidirectional endoscopies, as these require precise semantic distinction between the suggested recalls for the upper and lower endoscopic component. Although the model was able to correctly isolate, the colonoscopy-relevant recall text, we did find a failure in which the model misinterpreted a recall age as a recall interval (Figure 2C). This failure mode prompted a model update to GPT-4 Turbo, which demonstrated improved contextual reasoning and corrected the misinterpretation without regression in overall accuracy.

As one approach to more robustly handle this failure mode, we transitioned to GPT-4 Turbo, which demonstrated enhanced reasoning capabilities and successfully handled edge cases, including differentiating between target-age and interval-based recalls (Figure 2D). Retrospective validation confirmed that GPT-4 Turbo maintained the high inference accuracy seen with GPT-4.

### Expanded validation of GPT-4 turbo results

To derive a more precise estimate of model performance, we performed expanded validation on a held-out set of 326 patient notes. Clinician inter-rater agreement was *κ* = 0.985, confirming annotation consistency. GPT-4 Turbo performed with an F1 = 0.891 (95% CI, 0.650-0.917), Precision = 0.872 (95% CI, 0.639-0.917), and Recall = 0.917 (95% CI, 0.667-0.917) (Figure S2). In 2 validation cases, GPT-4 Turbo inferred spurious colonoscopy recall intervals (eg, 3 months or 7 years) from letters that did not contain an explicit colonoscopy recommendation. For instance, one letter listed multiple unrelated follow-up timelines (“EGD repeat in 2–3 years,” “liver ultrasound every 6 months,” “clinic follow-up in 3 months”), prompting the model to incorrectly attribute the 3-month interval to colonoscopy surveillance. This represents a contextual overgeneralization failure in which temporal cues from other procedures were misapplied to the colonoscopy domain.

### Expanded quality control including anomaly detection

As manual review becomes infeasible at larger scales, we implemented more efficient approaches for quality control at scale, namely field validation and anomaly detection. At intermediate scale (*N* = 1000), field validation–which allows rapid examination of the unique values for an output field rather than all values–identified instances where invalid values (“PARSE ERROR”) were returned often corresponding to patients who had aged out of surveillance), leading to further prompt revision.

**Figure 3.**
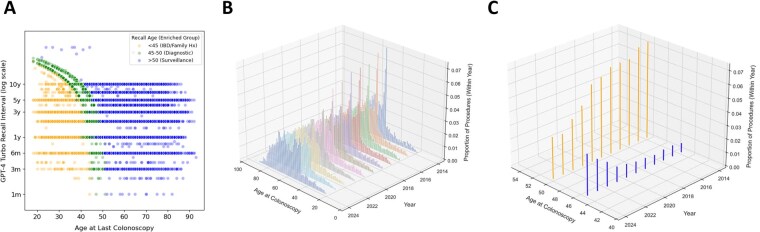
(A-C) Exploratory data analysis of population-wide colonoscopy recall dataset. (A) Comparison of calculated age at last colonoscopy and GPT-4 Turbo inferred recall interval. Points are colored by qualitative subgroup (yellow = next projected colonoscopy before age 45, including patients with IBD and family histories of CRC; green = patients projected to return for their next colonoscopy at a standard screening initiation age, enriched for diagnostic colonoscopies; blue = patients over age 50 with standard risk-based screening and surveillance intervals). (B) Distribution of age at last colonoscopy over time in our institutional cohort. (C) An increase in the proportion of patients being screened at age 45 coincides with the 2021 USPSTF guideline recommendation to move the age of screening initiation from 50 (orange) to 45 (blue). Original figure created by authors using data from hospital database (refer to methods section). Visualization designed using Adobe Illustrator.

To detect even lower frequency errors at scale, we computed derived features (eg, “age at recall,” “age at last colonoscopy”) and performed anomaly detection, in which we evaluated joint variable distributions for outliers (Figure 3A). At full scale (*N* = 118 181), we qualitatively identified outlier populations with unexpectedly short or long recalls, that is, below 6 months or over 10 years, respectively. Small-value outliers typically corresponded to clinic follow-ups rather than colonoscopy follow-ups, while large values in which a patient was projected to return well beyond a standard screening age typically represented misinterpretation of absolute recall year as recall interval. These quality control methods identified rare failure modes: ∼1 in 1000 (field validation) and ∼1 in 10 000 (anomaly detection).

### Comparison to rule-based NLP

To assess the performance of GPT-4 Turbo relative to historical standards for NLP tasks, we performed a comparison against a rule-based NLP pipeline implemented using spaCy v3 (see Methods). Across a comparison cohort of 21 172 patients, there was a relatively high concordance between the 2 approaches (96.1%). Among clinically significant discrepancies (ie, those exceeding 6 months; see Methods), the majority demonstrated an incorrect spaCy v3 inference in the setting of a correct GPT-4 Turbo inference (95.6%; Table S1). Based on this analysis, we estimate an absolute risk reduction in clinically significant inference errors of 3.39%, corresponding to over 3000 patients in our full cohort.

To better understand the errors during this inference task, we assessed the failure modes of these models based on the discrepant cases. In addition to the error modes described above for GPT-4 Turbo, we identified a set of 3 failure modes that captured nearly all of the spaCy v3 errors (Table S2). Inability to extract non-traditional recall representations (eg, age-based or date-based recalls) accounted for the largest proportion of misclassification (1.50% of the full cohort), followed by errors in range extraction (ie, mismapping a range to a single value; 1.46%), and incorrect contextual interpretation (eg, extracting a clinical follow-up interval instead of colonoscopy interval; 0.64%). Together, these reasoning-based limitations explained nearly all clinically meaningful discrepancies observed with rule-based extraction.

### Real-world deployment and usability

Following implementation of quality control filters as above, structured AI-generated recall recommendations were uploaded into the new EHR’s health maintenance tab along with a brief automated comment citing the source type (report vs letter) and date. This transparency enabled clinicians to quickly verify recall intervals and address discrepancies.

Post-deployment feedback from primary care physicians was collected by the IS team and was generally positive. One exception to this was a message inquiring about the absence of recall data for a patient whose procedure occurred at a low-volume affiliate—a known limitation of our pipeline anticipated to be quite uncommon across the cohort (<1% of procedures).

### Integrative analysis at scale

Given the unique opportunity to examine structured colonoscopy recall data for a massive clinical population, we explored what additional insights might be evident at scale. Further evaluation of groups in our anomaly detection plot identified 3 populations with differentially enriched groups (Figure 3A):

1. Patients aged below 45 with recall dates projected to be before a standard screening start, representing those with family histories of colon cancer or IBD who often require more frequent surveillance.^7^
2. Patients with abbreviated recall intervals and next colonoscopy recommended between ages 45 and 50, representing those who had diagnostic colonoscopies with plans to return for standard age-based screening.
3. Patients with next colonoscopies at ages beyond this range were primarily those in standard risk-adjusted screening and surveillance pathways.

In a complementary analysis, we assessed the distribution of ages for patients undergoing colonoscopies for each year in the cohort (Figure 3B). An increasingly prominent peak at age 45 could be appreciated relative to age 50 beginning in 2021 (Figure 3C), corresponding to the USPSTF guideline change that year.^8^ These findings—reflecting both patient and provider level trends across time—hint at the potential insights made possible by harnessing LLMs within a broader end-to-end pipeline to structure data at scale.

## Discussion

### Structured data transitions create critical vulnerability windows

A growing trend in healthcare involves the migration toward structured documentation systems—whether through individual health maintenance modules or full-scale EHR transitions. These results in a critical vulnerability window: the new fields may appear populated or reliable, yet important clinical information—such as prior surveillance recommendations—remains buried in unstructured notes. In this setting, clinicians may unknowingly omit essential tasks, assuming the structured data is accurate. These findings align with prior observations that structured-data migrations frequently overlook nuanced temporal recommendations embedded in narrative notes.^5^^,^^6^ Both studies demonstrated that automated colonoscopy-interval extraction is feasible but dependent on tightly templated source reports, highlighting why data fidelity often deteriorates during large-scale transitions involving heterogeneous formats. Although this implementation was motivated by an institutional EHR transition, the underlying extraction workflow may also be applicable to other scenarios where historical unstructured clinical documentation must be converted into structured data.

This implementation highlights one such example—colonoscopy recall intervals, which were inconsistently populated in structured fields during a major EHR overhaul, underscoring the risks of relying on default placeholders rather than individualized recommendations. Prior NLP-based efforts laid important groundwork for automating recall interval identification, but our implementation addressed a distinct operational challenge: restoring documented clinician recommendations during a system-wide EHR transition rather than prospectively determining guideline concordance. Prior to intervention, over 70% of recall intervals would have had placeholder values in the previously planned migration approach that were larger than the clinically indicated interval—a gap that would have introduced risk of missed surveillance and potential diagnostic delays (Figure S3).

### LLMs as transitional infrastructure in the era of structured data

Our pipeline was a multi-layered system designed to ensure fidelity and scalability (Figure 1). Although deployed in a batch mode during our institution’s EHR transition, the pipeline was built with transparent traceability in mind. Clinicians were able to view AI-extracted recall intervals alongside supporting text from the original reports, enabling real-time verification and clinical trust. This human-in-the-loop design supports not just migration, but ongoing quality assurance. Although demonstrated here using colonoscopy recall intervals, the underlying pipeline architecture—combining OCR preprocessing, LLM-based extraction, layered quality control, and structured EHR integration—may be adaptable to other structured data migration tasks involving historical unstructured clinical documentation.

Earlier NLP approaches—including rule-based, statistical, and hybrid methods—have been successfully applied to clinical information extraction tasks, including prior work on colonoscopy surveillance interval identification.^5^^,^^6^^,^^9^ In this study, the spaCy-based implementation was used as a practical baseline comparator for this specific migration task rather than as a comprehensive representation of traditional NLP. Within this workflow, the LLM-based approach appeared more robust to variable note structures, OCR-derived text, and contextual ambiguity. Expanded benchmarking suggested that, in this specific task, discrepancies in the rule-based baseline often involved contextual ambiguity or non-standard recall representations, such as age-based recommendations or interval ranges. Though infrequent globally (<5% of notes), the magnitude of these errors could lead to clinically significant delays in cancer-preventative care. Thus, for highly standardized formats in low-risk scenarios (ie, where errors are unlikely to have significant negative consequences for care), traditional rule-based approaches may be preferable given their efficiency and cost savings. On the other hand, for tasks in which information may be more variably represented and in which there may be substantial downstream consequences for errors in inference, LLM-based or combined implementations may be worthy of consideration. The performance results here suggest potential value to incorporating LLMs in larger real world clinical safety workflows, particularly when appropriately operationalized to enable structured, auditable outputs. It reconciles inconsistencies across diverse document types, supports structured field completion at scale, and maintains clarity for clinician oversight.

### System-level impact and cost avoidance

The full pipeline processed 118 181 records in approximately 9 hours (mean ≈ 2 seconds per record). Total compute expenditure was approximately $12 345 USD (LLM ≈ $4135; OCR ≈ $8210). In contrast, manual abstraction—estimated at 15 minutes per record—would have required ≈29 500 person-hours.^4^ This represents a potential > 400× efficiency gain relative to manual review.

To illustrate the potential downstream implications of restoring patient-specific recall intervals, we performed an exploratory modeling analysis based on previously published estimates of surveillance benefits and colorectal cancer costs. Under the assumptions of these prior epidemiologic models, replacing default 10-year intervals with patient-specific recommendations corresponded to an estimated reduction of up to 6092 colorectal cancer cases and 406 160 additional patient-years of surveillance (Figure S3).^10^^,^^11^ These figures should be interpreted as model-based projections rather than directly observed clinical outcomes. Using the same assumptions, projected cost avoidance ranged from approximately $400-670 million (Figure S3), with total inference and OCR expenditures of under $20 000 per pipeline execution.^12^ There may also be associated improvements in preventative health equity given that at-risk populations might be most vulnerable to loss to follow up in the setting of inappropriately long surveillance intervals.^13^

### End-to-end workflow integration

A key strength of our system is its integration of extraction, validation, and EHR-facing output within a single structured data backfill workflow. The output is integrated into physician-facing interfaces, allowing providers to trace each structured recall recommendation back to its original source note for validation. This design ensures transparency, supports clinical oversight, and lays out the foundation for future real-time or continuous NLP integration. Unlike prior NLP efforts focused solely on classification, our approach enables scalable chart abstraction with human-in-the-loop validation, making it well suited for multi-institutional deployment and continuous quality improvement. This workflow demonstrates how LLM-based migration can coexist with traditional NLP quality frameworks, combining semantic flexibility with traceable, auditable outputs that align with clinical governance standards.

### Limitations

Our study reflects a single academic medical center’s experience, potentially limiting generalizability. Unlike earlier NLP efforts designed for surveillance-guideline inference, our system focused exclusively on accurate data migration during an EHR transition, so guideline concordance was outside scope. In addition, the downstream clinical and economic analyses were exploratory model-based projections derived from published estimates and were not directly measured outcomes within this implementation study. We also did not perform external validation on datasets from other institutions, and therefore the model’s performance across alternative EHR structures and documentation styles remains to be determined. Our legacy EHR (webOMR) was one of the earliest systems developed, so pre-processing challenges might differ in alternative systems.^14^ We relied primarily on GPT-4/GPT-4 Turbo; newer reasoning enabled models might improve accuracy but increase costs.^15^ Finally, while we executed 2 batch processes at scale, we did not deploy a prospective recall system, which could surface additional implementation challenges (eg, real-time access, fault tolerance redundancy, and proactive patching). Post-deployment performance data were limited to early field validation and anomaly-detection audits, so long-term monitoring and user acceptance metrics remain areas for future study.

## Conclusion

Our LLM-driven colonoscopy recall system demonstrates how modern tools can bridge this gap when deployed within broader end-to-end pipelines and underscores the potential of transformer-based architectures to bring unprecedented levels of precision to diverse aspects of clinical care, building upon decades of NLP research to enable practical, real-world data migration.

## Supplementary Material

ooag070_Supplementary_Data

## Data Availability

The software implementation used in this study is publicly available at: https://github.com/ai-safety-public-repo/colonoscopy_recall.
