# Supplementary material for: Design and implementation of an end-to-end AI-driven colonoscopy recall workflow at scale
Source: JAMIA Open. 2026 May 6;9(3):ooag070. doi: 10.1093/jamiaopen/ooag070 (PMC13148243; doi:10.1093/jamiaopen/ooag070)
Supplement: ooag070_Supplementary_Data [file ooag070_supplementary_data.zip › Supplementary Legend with Captions and Figures-2-2.docx]

**Supplementary Legend:**

**Supplementary Figure S1. Evolution of a prompt: annotated stages of prompt development for colonoscopy recall inference.**


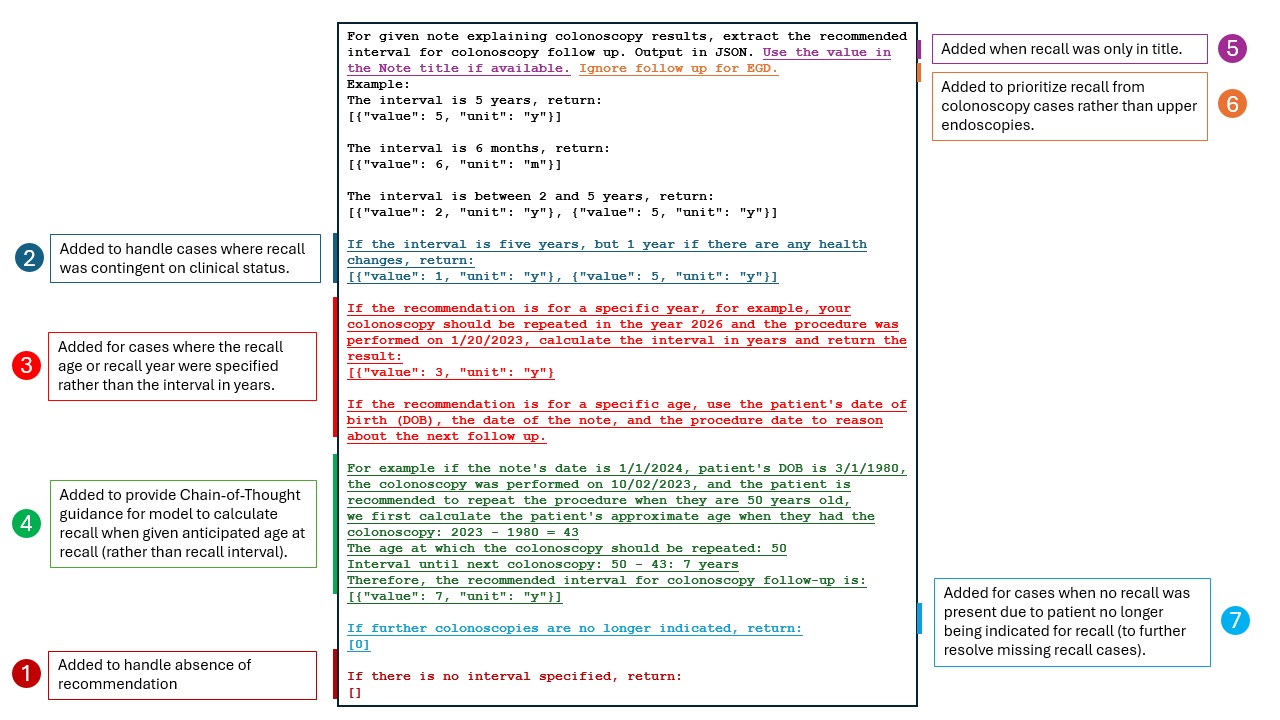


**Supplementary Figure S1. Evolution of a prompt: annotated stages of prompt development for colonoscopy recall inference.**

The iterative evolution of the prompt inference is shown here, with the base prompt shown in black, and subsequent additions shown in distinct colors along with underlining for emphasis. Clinical context/motivation for each update is shown in the annotations in the margins. Initial QC (N=100 patients) was assessed with prompt revision 2, with subsequent additions following later rounds of QC. Original figure created by authors using data from hospital database (refer to methods section). Visualization created in Microsoft PowerPoint.

### **Supplementary Figure S2. Comparative Performance of GPT-4 Turbo and Rule-Based NLP on Colonoscopy Recall Extraction**


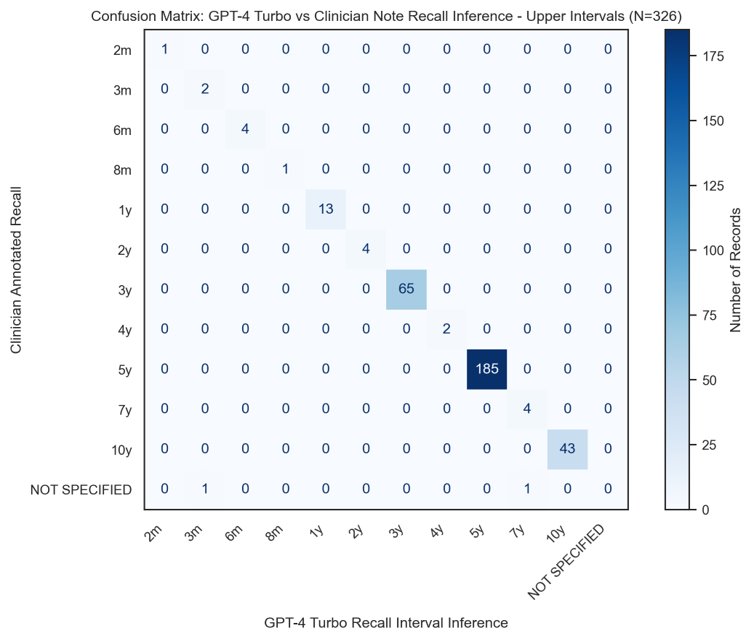


### **Supplementary Figure S2. Comparative Performance of GPT-4 Turbo and Rule-Based NLP on Colonoscopy Recall Extraction**

Confusion matrix comparing GPT-4 Turbo–inferred recall intervals with clinician-annotated references (N = 326 patient letters). Most predictions aligned with clinical review, though in two cases GPT-4 Turbo generated spurious intervals (e.g., 3 months or 7 years) despite no colonoscopy recall being documented, reflecting contextual overgeneralization from unrelated follow-up phrases (e.g., “EGD repeat in 2–3 years,” “clinic follow-up in 3 months”).

**Supplementary Figure S3. Potential impacts of an LLM-derived recall inference workflow on long term risk reduction and downstream cost savings relative to a non-individualized 10-year recommendation.** **Supplementary Figure S3. Potential impacts of an LLM-derived recall inference workflow on long term risk reduction and downstream cost savings relative to a non-individualized 10-year recommendation.**


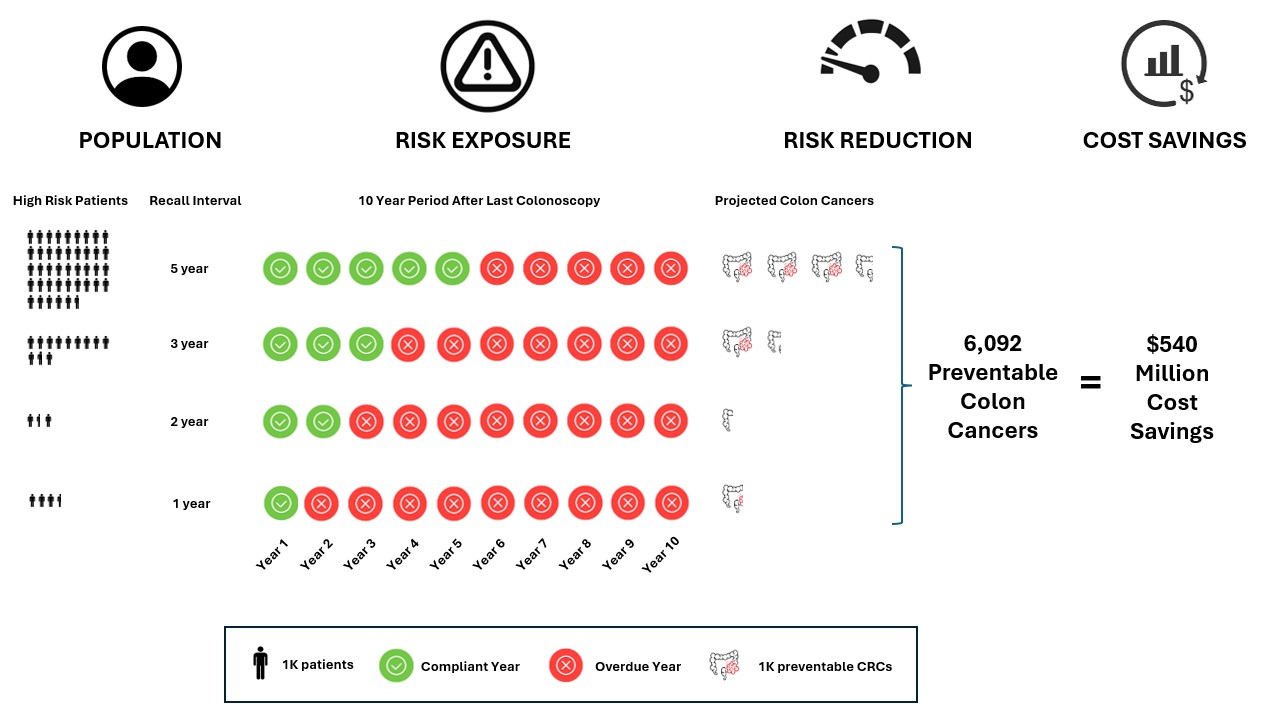


**Supplementary Figure S3. Potential impacts of an LLM-derived recall inference workflow on long term risk reduction and downstream cost savings relative to a non-individualized 10-year recommendation.** **Supplementary Figure S3. Potential impacts of an LLM-derived recall inference workflow on long term risk reduction and downstream cost savings relative to a non-individualized 10-year recommendation.**

Patients were considered high-risk if their individualized recall recommendation from their last colonoscopy was 5 years or less (so less than e.g., a standard 10-year recommendation). Each year of the following decade after the last colonoscopy is annotated based on whether a patient assigned to a default 10-year interval would be compliant with the last recommendation or not. Projected colon cancers for each risk group are displayed (using a baseline 2% / year rate of CRC in overdue patients and assuming an efficacy of 75% for colonoscopy).^6,7^ An average cost of CRC management of $80,000/patient was used to model total cost savings.^12^ Original figure created by authors using data from hospital database (refer to methods sections), with parameters adapted from Sullivan et al. (2022)⁶, Chen et al. (2003)⁷, and Yabroff et al. (2008)⁸. The figure was created using Adobe Illustrator.

**Supplementary Table S1. Clinically significant recall misclassification rates and clinical risk reduction by extraction method.**

| **Metric** | **Value** |
| --- | --- |
| spaCy v3 error rate | 3.60% |
| GPT-4 Turbo error rate | 0.21% |
| Absolute risk reduction | 3.39% |
| Relative risk reduction | ~94% |
| Prevented clinically significant errors  (in ~100k pt cohort) | ~3.6k patients |

**Supplementary Table S1. Clinically significant recall misclassification rates and clinical risk reduction by extraction method.**
Discrepancies were stratified by clinical severity based on deviation from clinician-documented surveillance recommendations (moderate >6 months to ≤2 years; major >2 years). GPT-4 Turbo demonstrated correct recall timing across all moderate-severity strata and in the majority of highest-severity cases examined, resulting in substantial reduction of clinically meaningful surveillance misclassification relative to the rule-based spaCy v3 baseline.

**Supplementary Table S2. Dominant spaCy v3 failure modes in clinically significant recall discrepancies.**

| **Failure Mode** | **Description** | **Representative Example** | **Estimated Overall Rate** |
| --- | --- | --- | --- |
| **Age-based / date arithmetic failure** | Inability to compute recall timing anchored to patient age or future date | “Repeat at age 50” → SpaCy outputs fixed interval rather than years-to-target | **1.50%** |
| **Range or conditional collapse** | Flattening of interval ranges to a single value | “Repeat in 3–5 years” → extracted as “3 years” | **1.46%** |
| **Context confusion** | Extraction of clinic follow-up or non-recall timing instead of surveillance recommendation | “RTC in 3 months” interpreted as colonoscopy recall | **0.64%** |

**Supplementary Table S2. Dominant spaCy v3 failure modes in clinically significant recall discrepancies.**

SpaCy extraction errors within moderate and major deviation strata were manually reviewed and categorized into three primary failure modes: age-based or date-dependent arithmetic failure, interval range or conditional collapse, and context confusion. Failure mode frequencies were estimated using stratified bin-weighted sampling based on each stratum prevalence in the full cohort.
